# ID 254 - Impacto de Diferentes Coberturas Vacinais da Vacina de Alta Dosagem versus Vacina de Dose-Padrão contra o Vírus Influenza em Idosos no Brasil

**DOI:** 10.5327/2237-9622.2025.v34s1.254

**Published:** 2025-11-25

**Authors:** Bianca Caroline Salvador, Sarah Watanabe, Bernardo Salustio Pires, Frederico Magro, Aline Barbosa, Alexandre Taminato, Gerald Moncayo, José Cassio de Moraes, Rosana Richtmann

**Keywords:** vacina, influenza, cobertura vacinal

## Abstract

**Introdução:**

O vírus influenza causa um grande impacto sobre a saúde dos idosos. As consequências da influenza são amplas, acarretando ônus para além da doença respiratória, sendo relacionada a altas taxas de hospitalizações, agravamento de doenças crônicas, declínio funcional, pneumonia e aumento da mortalidade. Atualmente, o Programa Nacional de Imunização (PNI) oferece a todos os idosos imunização com vacina trivalente de dose-padrão (SD-TIV – do inglês, ), entretanto a cobertura vacinal vem sofrendo uma tendência de declínio. Sabe-se que a cobertura obtida com campanhas de vacinação não depende apenas da disponibilidade e da divulgação das vacinas, mas também da aceitação e da conscientização do paciente a ser imunizado. Assim, mesmo na presença de suficiente oferta de doses, a proteção vacinal no Brasil tem enfrentado desafios. Nesse sentido, uma alternativa à busca de aumento da cobertura é o uso de imunizantes de maior eficácia, como vacinas de alta dosagem (HD-TIV – do inglês, ). O objetivo do estudo é avaliar como o uso de HD-TIV poderia impactar a proteção de pacientes com mais de 60 anos de idade, destacando pacientes acima de 80 anos no Sistema Único de Saúde (SUS).

**Método:**

Um modelo econômico estático de árvore de decisão desenvolvido anteriormente para análise de custo-utilidade de HD-TIV foi usado com o objetivo de comparar diferentes coberturas vacinais das tecnologias disponíveis para profilaxia da influenza em idosos, HD-TIV versus SD-TIV. A eficácia das vacinas foi baseada em estudos publicados, e os riscos de desfechos foram estimados com base em dados históricos do Departamento de Informação e Informática do Sistema Único de Saúde (DataSUS). Como cenário atual, o número de hospitalizações e óbitos por influenza foi calculado relativo à população e à cobertura vacinal obtida no Brasil em 2022 com o uso da vacina SD-TIV, atualmente empregada no SUS, valores obtidos do Instituto Brasileira de Geografia e Estatística (IBGE) e DataSUS, respectivamente. O número de casos, hospitalizações e óbitos com uso de HD-TIV foi estimado com base na eficácia vacinal relativa incremental desse imunizante em relação à SD-TIV. Por fim, foi realizada uma análise de equivalência de cobertura vacinal necessária para chegar aos mesmos resultados clínicos estimados para HD-TIV com o uso de SD-TIV.

**Resultados:**

De acordo com o modelo, para os pacientes acima de 60 anos de idade com base em uma cobertura vacinal de 65,4%, se toda essa população fosse imunizada com uma vacina HD-TIV, em vez de uma vacina SD-TIV, poderiam ser evitadas 22.282 internações hospitalares relacionadas à influenza, 78.115 internações hospitalares por causas cardiorrespiratórias potencialmente atribuíveis à influenza e 10.795 óbitos, resultado que equivaleria a vacinar 95,5% da população com mais de 60 anos no SUS com SD-TIV. Entre os idosos acima de 80 anos, a imunização de 63,9% da população com HD-TIV acarretaria a prevenção de 9.366 hospitalizações apenas relacionadas à influenza, 23.711 internações cardiorrespiratórias e 4.557 óbitos evitados, resultado que equivaleria a vacinar 93,4% da população com mais de 80 anos no SUS com SD-TIV.

**Conclusão:**

Com a tendência de declínio das taxas de cobertura vacinal no Brasil nos últimos anos e considerando-se a dificuldade e o esforço de sua retomada, o uso de vacinas com maior eficácia, como a HD-TIV, pode garantir melhor proteção dos idosos contra infecções por influenza e suas complicações, o que potencialmente pode ser uma melhor escolha de saúde pública.

